# A new conceptional model for deriving average dermal absorption estimates from studies with multiple tested concentrations for non-dietary risk assessment of pesticides

**DOI:** 10.1007/s00204-022-03320-3

**Published:** 2022-06-15

**Authors:** Felix M. Kluxen, Edgars Felkers, Steve McEuen, Philip Fisher, Christian Strupp, Christine Lorez, Jeanne Y. Domoradzki, Christiane Wiemann

**Affiliations:** 1 ADAMA Deutschland GmbH, Edmund-Rumpler-Str. 6, 51149 Cologne, Germany; 2 FMC Corporation, Philadelphia, PA USA; 3 Crop Science Division, Bayer SAS, Sophia Antipolis, France; 4 Gowan Crop Protection Ltd., Reading, UK; 5 Syngenta Crop Protection AG, Basel, Switzerland; 6 Corteva Agriscience, Indianapolis, IN USA; 7 BASF Oesterreich GmbH, Vienna, Austria

**Keywords:** Dermal absorption, Exposure assessment, Risk assessment, Pesticides, Plant protection products, Simplified risk assessment

## Abstract

Dermal absorption values are used to translate external dermal exposure into potential systemic exposure for non-dietary risk assessment of pesticides. While the Environmental Protection Agency of the United States of America (US EPA) derives a common dermal absorption factor for active substances covering all related products, the European Food Safety Authority (EFSA) requests specific product-based estimates for individual concentrations covering the intended use rates. The latter poses challenges, because it disconnects exposure dose from applied dose in absorption studies, which may not be suitable in scenarios where concentration is not relevant. We analyzed the EFSA dermal absorption database, collected 33 human in vitro studies from CropLife Europe (CLE) companies, where ≥3 in-use dilution concentrations were tested, and 15 dermal absorption triple pack datasets. This shows that absolute dermal absorption correlates with absolute applied dose on a decadic logarithm-scale, which is concordant with the toxicological axiom that risk is driven by exposure dose. This method is radically different from the current European approach focused on concentrations and offers new insights into the relationship of internal and external exposure doses when utilizing data from in vitro studies. A single average dermal absorption value can be simply derived from studies with multiple tested concentrations, by calculating the y-intercept of a linear model on a decadic logarithm scale while assuming a slope of 1. This simplifies risk assessment and frees resources to explore exposure refinements. It also serves as a basis to harmonize dermal absorption estimation globally for use in exposure-driven risk assessments.

## Introduction

Dermal absorption values are used to translate external dermal exposure into potential systemic exposure for risk assessment. Predominantly, in vitro studies using human skin and conducted according to OECD TG 428 (OECD 2004) are used for pesticide and biocide registrations. In Europe, this was driven by guidance documents published by EFSA (EFSA 2012, 2017). The US EPA also committed to reduce animal tests in general (US EPA 2019) and thus in vitro dermal absorption methods gain support (Allen et al. 2021). Similar changes occur in other countries globally, such as Australia (APVMA 2019), Brazil (ANVISA 2019; CONCEA and MCTI 2014), Canada (PMRA 2016), China (China 2017), Japan (JMAFF 2019) and South Korea (So et al. 2014). However, dermal absorption evaluation criteria vary between authorities (Plaza et al. 2021).

US EPA generally assumes a single representative dermal absorption value for an active ingredient independent of its use scenario (US EPA 2007). This may be viewed as deriving an average value as an active ingredient property, similar to oral absorption, covering uses for all products containing the respective active.

EFSA assumes a dermal absorption value depending on the concentration of the in-use dilution of a specific use for a specific product. EFSA applies much scrutiny to the in vitro dermal absorption assay evaluation, see EFSA dermal absorption guidance (EFSA 2017), to avoid hypothetical under-prediction of absorption. EFSA’s dermal absorption estimate derivation rules have been reviewed elsewhere (Kluxen et al. 2021) and in practice only increase study-derived values without intending to derive realistic absorption estimates. True dermal absorption under-prediction is very unlikely based on the assay design and conduct itself because it only considers cumulative dermal penetration and no other toxicologically relevant absorption, distribution, metabolism and excretion (ADME)/kinetic or dynamic processes. No other endpoint used in risk assessment is derived in such a scrutinous manner.

Absorption could be described in a very simple model as penetration probability, i.e., the probability of a single molecule to penetrate in a given timeframe. Let there be two hypothetical extreme cases as thought experiments. Case 1, assuming four doses of 1, 2, 10 and 100 molecules and that the probability of the molecule nearest to the penetration barrier penetrate is 1 and similar between the doses; the probability of other molecules penetrating is 0. Then, the relative absorption of the doses after the observation time will be 100, 50, 10 and 1%, respectively. As the other extreme, Case 2, assume four doses of 10, 20, 100 and 1000 molecules and each molecule in the dose has the same probability of 0.1 to penetrate. Then the resulting relative absorption values are 10% for all doses. In practice, there will be a probability gradient over the molecules depending on their distance to the penetration barrier; there is further an interaction of steric interference, Brownian motion and Fick's laws, and for agrochemical formulations also other aspects, see Discussion. However, the simple model explains how the relative dermal absorption value can be affected by concentration or dose. The model may also allow us to assess whether data correlates more with the one or the other hypothetical case.

If an exposure scenario considered in risk assessment is evaluated based on a sprayed concentration and the concentration is outside the tested concentration range, EFSA guidance suggests increasing tested dermal absorption values pro-rata, independent of the actual exposure dose or scenario. For example, if a tested concentration of 1 g/L has a relative absorption of 1%, but the sprayed concentration is 0.1 g/L, the dermal absorption value is generically considered to be 10%.

There are thus challenges in non-dietary risk assessment as exposure calculations consider dose per cm^2^ exposed skin surface or dose per kg body weight, while dermal absorption estimates as applied in the risk assessment formula consider the fraction absorbed based on the tested concentration, *i.e.*, the relative dermal absorption value (DA_rel_). Hence, the dermal absorption estimate is disconnected from the exposure dose, especially in cases where exposure is not directly to a spray dilution but rather to dried or partly remoistened residues. This results in unrepresentative assessments, for example for re-entry workers. While corresponding dermal absorption tests can be adapted for re-entry scenarios (Aggarwal et al. 2019; Morgan et al. 2020), those are rather complex and depend on the specific exposure scenario.

The key idea for the current manuscript was to hypothesize that the absolute amount applied may be a good characteristic of the absolute amount absorbed. The subsequent idea was realizing that the approach also allows the derivation of an average relative dermal absorption value from dermal absorption studies conducted with multiple concentrations. Thus, exposure calculations could be tremendously simplified as the dermal absorption value remains constant and independent of exposure dose, while potential systemic exposure depends only on exposure dose and not assumed and hypothetical exposure concentration. This approach offers a tool to discuss the relationship of external and internal exposure dose and may also facilitate harmonization of US and European assessment criteria.

## Methods

Mean human in vitro data from the EFSA dermal absorption database (EFSA DA DB) were selected based on the criteria presented in Kluxen et al. (2019); further “Wax block” and “Pasta bait” formulation types were excluded. Mean human in vitro data using studies with multiple concentrations and mean rat in vitro and in vivo data and corresponding human in vitro data were collected from CLE member companies in a Microsoft Excel (Redmond, USA) sheet. The data were transferred into the free statistical software R (R Core Team 2020) using RStudio (RStudio Team 2019) and *readxl* (Wickham and Bryan 2019). The relationship of the applied dose in µg/cm^2^ and the absolute amount absorbed in µg/cm^2^ applied was explored by graphical analysis using *ggplot2* (Wickham 2016). Statistical analyses were conducted with the R base/stats package or the *lme4* function to fit a mixed-effects model considering “Product” as a random effect (Bates et al. 2015).

Linear models were fitted on decimal logarithm (log10)-transformed applied dose and absolute amount absorbed, with the following formula:

$$\log 10\left( {\text{absorbed dose}} \right) \, = {\text{ slope}} \times \log 10\left( {\text{applied dose}} \right) \, + \, y{\text{ - intercept}}$$

EFSA previously noted that dermal absorption data may be well fitted to a logit model for the derivation of default values (EFSA 2017), which was also observed during the preparation of Aggarwal et al. (2015), who log-transformed the data for statistical analysis (the publication shares co-authors with the current manuscript). The approach was, however, previously not suggested for the evaluation of individual dermal absorption studies. According to EFSA, “The logit transformation is essentially indistinguishable from log-transformation when the fraction absorbed is small but stretches the scale out better for higher levels of absorption.”

Dermal absorption was defined for the in vitro assays as the amount recovered in receptor fluid (RF) or amount recovered in receptor fluid and skin, including *stratum corneum* with the exception of the first two tape strips, which is often called “potentially absorbed dose”.

**Fig. 1:**
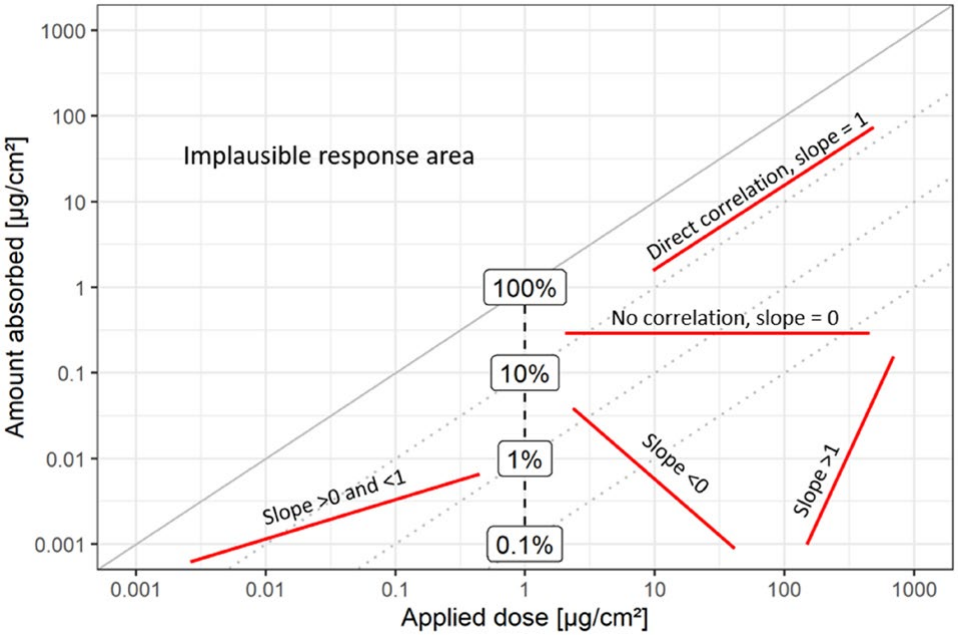
Log–log plot of amount absorbed to applied dose in µg/cm^2^ and potential response patterns. Red lines indicate hypothetically fitted linear models of dermal absorption data with different slopes. The labels indicate relative absorption (in %) that relate to the *y*-axis intercept of a linear model, *i.e.*, at *x* = log10(0) = 1, indicated by the vertical dashed black line (color figure online)

The graphical analysis utilizes a “log–log” two-dimensional scatter plot, see Fig. 1. Here, the absolute absorbed amount in µg/cm^2^ applied is plotted against the applied dose in µg/cm^2^ and the graph axes are scaled as log10. If the amount applied is similar to the amount absorbed, this corresponds to 100% relative dermal absorption, which is shown by a solid identity line, which has a slope of 1. Dotted lines with the same slope, *i.e.* “isoslopes”, but with y-axis intercepts spaced in tenfold distances, indicate relative absorption values of 10, 1 and 0.1%.

Linear models can be fit to the dermal absorption data and their slopes visually assessed. When the fitted lines are parallel to the isoslopes, absorption directly correlates with the applied dose on a log10-scale. Here, the relative dermal absorption value is given by the *y*-intercept (which is at *x* = 1 on log scale). If the slopes of the fitted lines are not parallel to the isoslopes, relative dermal absorption changes with the applied dose. This may be due to a dependence on applied concentration or other physical–chemical effects, see “Discussion” section. If a slope of 1 is assumed for such a case, the y-intercept will represent an average dermal absorption value. There is a dedicated section below on the derivation of average dermal absorption values with practical examples.

Table 1 shows potential interpretations of the derived slope, assuming recovery relates to the applied test item. One key issue in dermal penetration studies is that other ADME processes are not considered. First, because only cumulative penetration is assessed and further only the recovery of the (typically) radiolabeled compound equivalent is assessed independently of whether the amount penetrated corresponds to the original radiolabelled compound or degradates/metabolites thereof (Kluxen et al. 2021). The in vitro studies ignore any excretion kinetic which is in counterbalance to the penetration. The cases in Table 1 indicate, that the slope approach for interpretation is abstract especially for in vitro studies, where ADME processes play a limited role. However, the slope approach is instructive when considering dermal absorption data for risk assessment. While a slope of 1 is directly linked with the toxicological axiom of dose driving risk, it is implausible that ADME processes would not affect toxicity. It is also implausible that ADME always increases or decreases toxicity. Thus, the studies only show one aspect of ADME upon dermal exposure and are a gross abstraction themselves. On average one could assume that toxification and detoxification via ADME balances out, while it is often observed that ADME reduces risk. Hence, assuming a slope of 1 is on average conservative. A slope of 0 would indicate that the applied dose has no effect on dermal absorption, which is equivalent to the pro-rata approach considered by EFSA DA guidance documents.

**Table 1:** The “slope approach”: potential interpretation of slopes from linear models of dermal absorption data assuming recovered radioactivity relates to exposure towards the intact test item

Interpretation	Slope				
	> 1	1	> 0 and < 1	0	< 0
Absolute dermal penetration	Decreases with decreasing doses			Constant and independent of dose (EFSA pro-rata approach)	Increases with decreasing doses / inverse correlation
	Overly	Directly	Less than		
	dose-proportional				
Relative Dermal penetration	Decreases with decreasing dose	Constant and independent of applied dose	Increases with decreasing dose		
			< dose-proportional	dose-proportional	> dose-proportional
Small dose extrapolation limit in deterministic exposure calculation	Intersection with abscise, *i.e.,* 0% absorption → Very small doses are not absorbed	Constant	Intersection with identity line, *i.e.*, 100% absorption →Very small doses are completely absorbed		
*Assuming the in vitro penetration assay is modelling actual in vivo absorption*					
(A)DME processes (in vivo)	Reduce exposure/toxicity at lower applied doses	Do not affect exposure/toxicity	Increase exposure/toxicity at lower applied dose		
Predicted internal exposure	Smaller doses result in less systemic exposure than higher doses			All applied doses result in same systemic exposure	Lower applied doses result in higher systemic exposure
Biological plausibility	Plausible			Implausible	

Hence, when the selected data is plotted in the log–log plot and interpreted with the slope approach, the exercise may be instructive for risk assessment and management.

## Results

In the following, the data obtained from the EFSA DA DB and CLE are assessed by the log–log plot and fitted linear models. The results are shown in the chronological order of hypothesis generation, exploring the hypothesis in studies with multiple concentrations and in vivo/triple pack data.

### EFSA DA DB

**Fig. 2:**
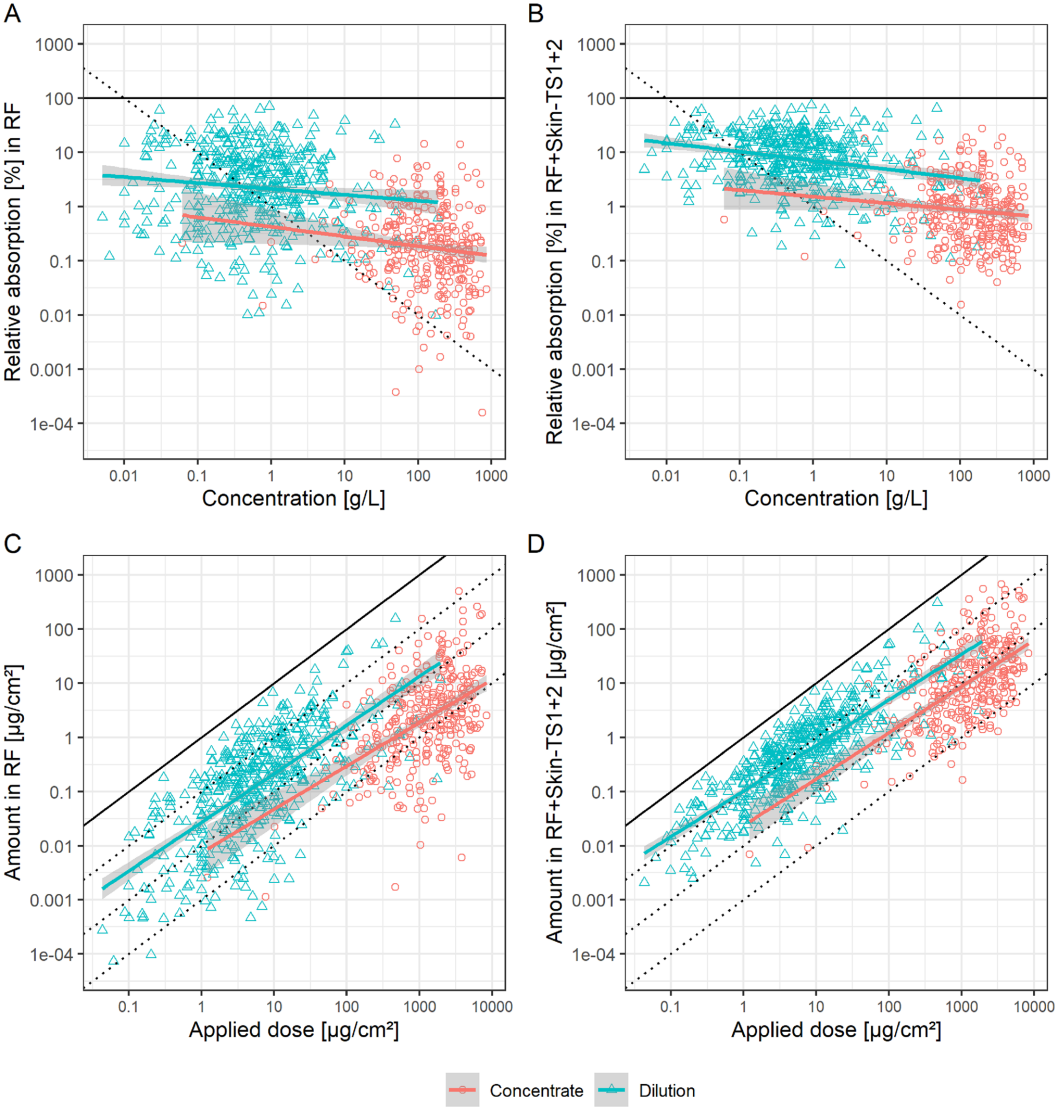
Mean data of the EFSA DA DB expressed as relative absorption and concentration in **A** receptor fluid and **B** amount in receptor fluid and skin minus tape strips 1 and 2 or expressed as absolute amounts (**C**) in receptor fluid or **D** potentially absorbed dose. Overplotted are linear models stratified by concentrate (red) or dilution (blue) (color figure online)

Figure 2 shows the mean dermal absorption data from the EFSA DA DB. The dataset comprises a large range of very different products.

Figure 2A, B show the data as currently used and assessed in Europe (however, on log-transformed scale), *i.e.*, relative absorption is assumed to be driven by concentration

The slope of the fitted models of the in-use dilutions is slightly negative, *i.e.*, −0.28 and −0.30 with *r*-squares of 0.01 and 0.04 for receptor fluid and potentially absorbed dose, respectively. The current pro-rata assumption in the EFSA guidance assumes a slope of −1, as indicated by the black dotted line in the plot background, *i.e.*, a direct inverse correlation of relative absorption and concentration. The graphical display indicates that the linear model substantially deviates from this assumption, thus, the hypothesis was formed that dose could be a better correlating predictor and accordingly investigated in Fig. 2C, D. Here, absolute absorption is plotted against absolute applied dose, as explained in the methods. While Fig. 2A, B (current EFSA method using concentration) do not show a good correlation, Fig. 2C, D (using dose) show an almost direct correlation. For the in-use dilutions, the slopes are 0.89 and 0.88, for receptor fluid and potentially absorbed dose, respectively, while *R*^2^s are 0.47 and 0.64. For the concentrates, the slopes are 0.78 and 0.84, for receptor fluid and potentially absorbed dose, respectively, while *R*^2^s are 0.21 and 0.32. Hence, both slope and variance appear to be better explained by dose than by concentration and the effect is more convincing for the in-use dilution data. The reason for the latter may be related to a higher influence of co-formulations for concentrated products. When the models (relative absorption ~ concentration *vs* absolute absorption ~ absolute dose applied) are compared by the Akaike information criterion (AIC), this is further substantiated (where lower numbers indicate a relative better model quality), see Table 2. For the in-use dilutions, the AIC decreased by about 968 for both receptor fluid and potentially absorbed dose, when using absolute values instead of relative absorption and concentration. The effect is slightly decreased for the concentrations, but still dramatic with a substantial decrease of about 669. Accordingly, this comparison indicates that a model using absolute dose applied and absolute absorption should be favored.

**Table 2:** Model fit comparison using slope, *R*^2^ and Akaike information criterion of the various possible models with in-use dilutions

	EFSA DA DB			CLE DB			CLE DB by product			
	Slope	*R*^2^	AIC	Slope	*R*^2^	AIC	Slope^a^	*R*^2^m	*R*^2^c	AIC
Relative DA RF ~ Concentration	−0.28	0.01	2261	−0.32	0.10	270	−0.35	0.12	0.89	155
Absolute DA RF ~ Applied dose	0.89	0.47	1294	0.68	0.35	270	0.64	0.32	0.92	154
Relative DA Pot ~ Concentration	−0.30	0.04	1828	−0.25	0.13	183	−0.31	0.19	0.83	108
Absolute DA Pot ~ Applied dose	0.88	0.64	860	0.76	0.59	183	0.69	0.55	0.91	107

R^2^m = marginal variance of the fixed effects

R^2^c = conditional variance of the full model including random effects

DA = Dermal Absorption

RF = Receptor Fluid

Pot = Potentially absorbed dose

^a^For fixed effects

The slopes of the fitted models using dose and absolute amount absorbed are close to 1, which indicates almost a direct proportional relationship of applied and absorbed dose. The 95% confidence intervals of the in-use dilution slopes for receptor fluid and potentially absorbed dose, *i.e.*, [0.81, 0.97] and [0.82, 0.93], respectively, do not include 0, which means that the EFSA DA DB provides evidence against the appropriateness of the pro-rata assumption for the EFSA dermal absorption guidance (EFSA 2017); compare the methods section for interpreting the slope in the log–log plot. In fact, the effect sizes are very close to 1 and further support a more direct relationship of dose and absorption than a direct relationship of concentration and relative absorption.

**Fig. 3:**
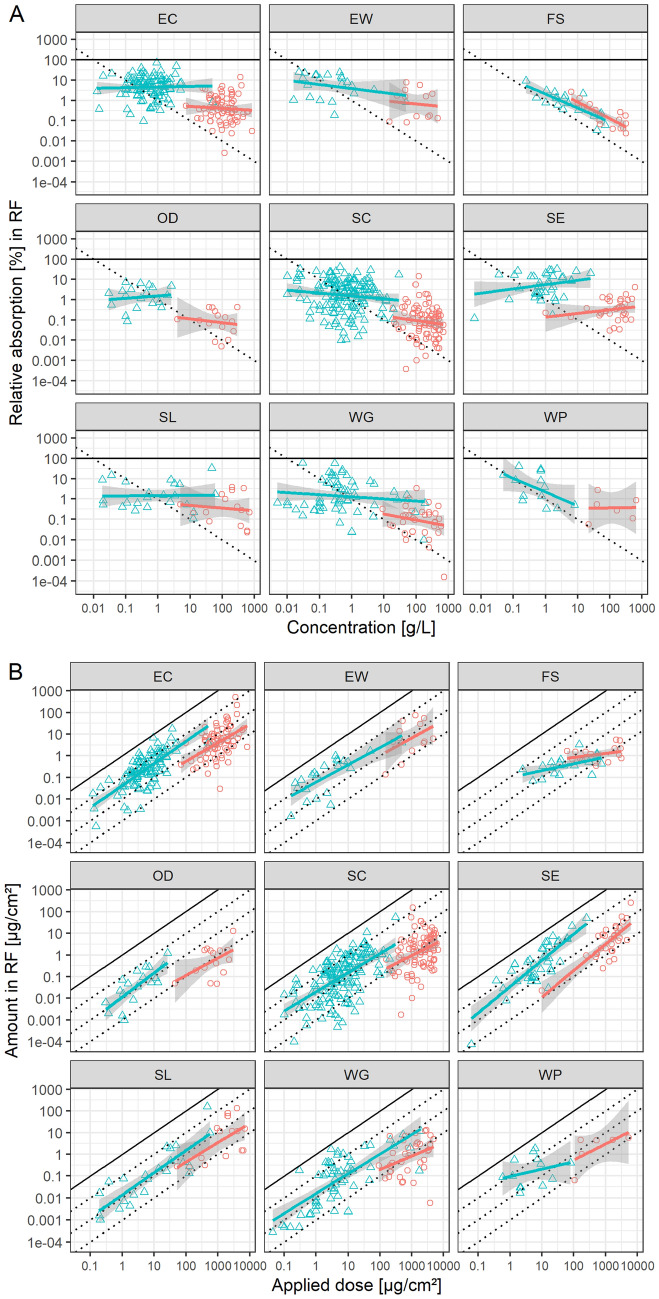
Mean data of the EFSA DA DB in receptor fluid either expressed as **A** relative absorption and concentration or **B** applied dose, stratified by formulation types (only contains formulation types with multiple studies available), using international formulation codes. Overplotted are linear models stratified by concentrate (red) or dilution (blue) (color figure online)

Figure 3 shows the data stratified by formulation types, using international formulation codes (CropLife International 2017; WHO 2016). All show similar dose–response patterns, except FS and WP.

When ignoring formulation type, the very simple linear model explains up to 64% of the observed variation in the data for the mean potential absorption data of the in-use dilutions, independent of any other parameters.

The data suggest that dermal absorption is well described by exposure dose, independent of the tested concentration. Further, this relationship appears to be almost constant. According to the potential interpretations in Table 1, the slope < 1 would indicate, if one transferred this to the in vivo scenario, that ADME effects increase internal exposure. However, this is most likely an artefact from the in vitro assay system, as excretion processes are ignored.

### Studies with multiple concentrations

While the data of the EFSA DA DB supports the hypothesis of favoring dose over concentration when characterizing dermal absorption properties, the model is rather naive by averaging over all available studies.

**Fig. 4:**
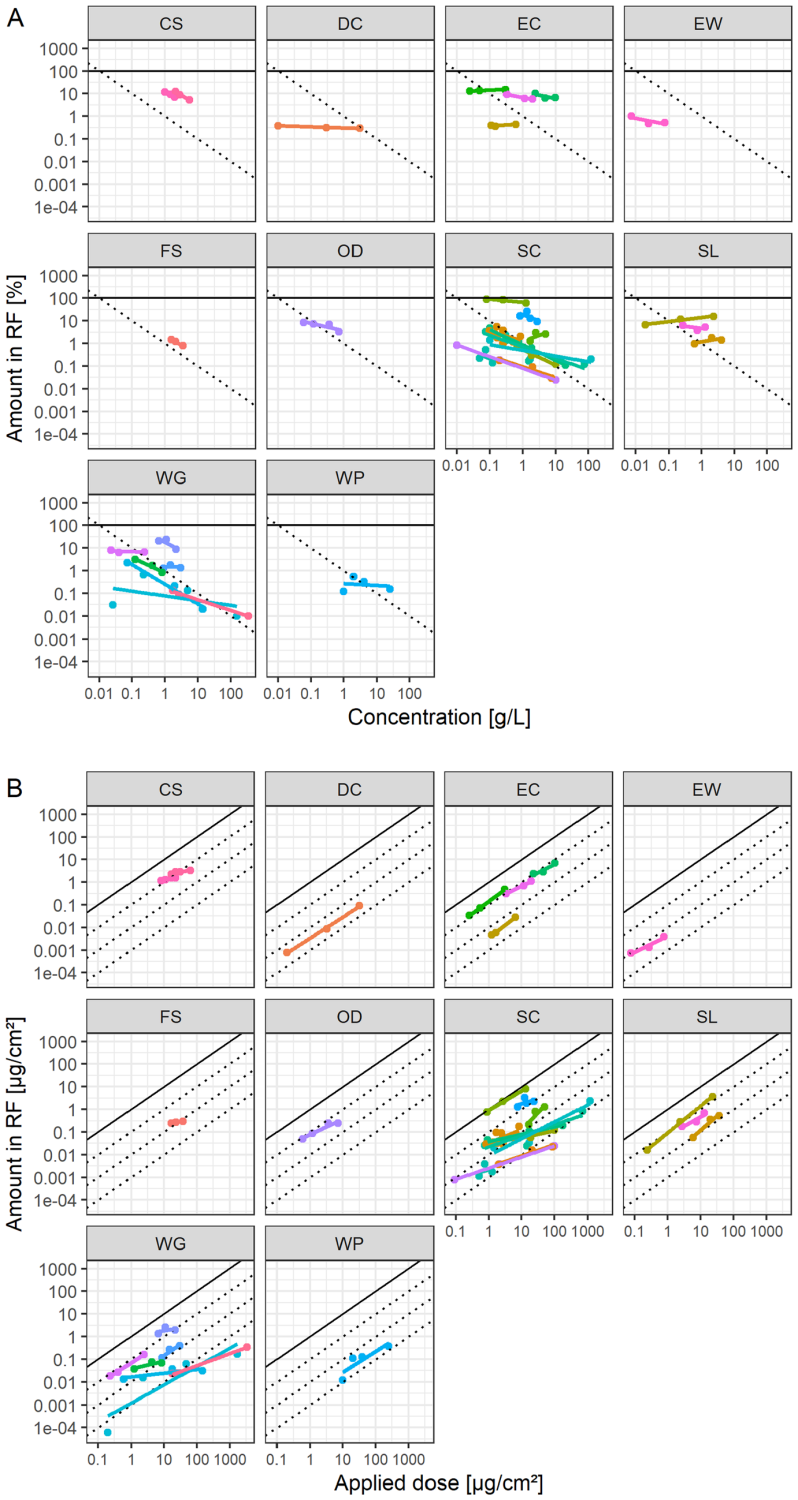
Receptor fluid recovery of CLE collected mean data of studies conducted with multiple concentrations of in-use dilutions, expressed as **A** relative absorption and concentration or **B** applied dose, latticed by formulation type. Linear models were fitted per study, colored by study

**Fig. 5:**
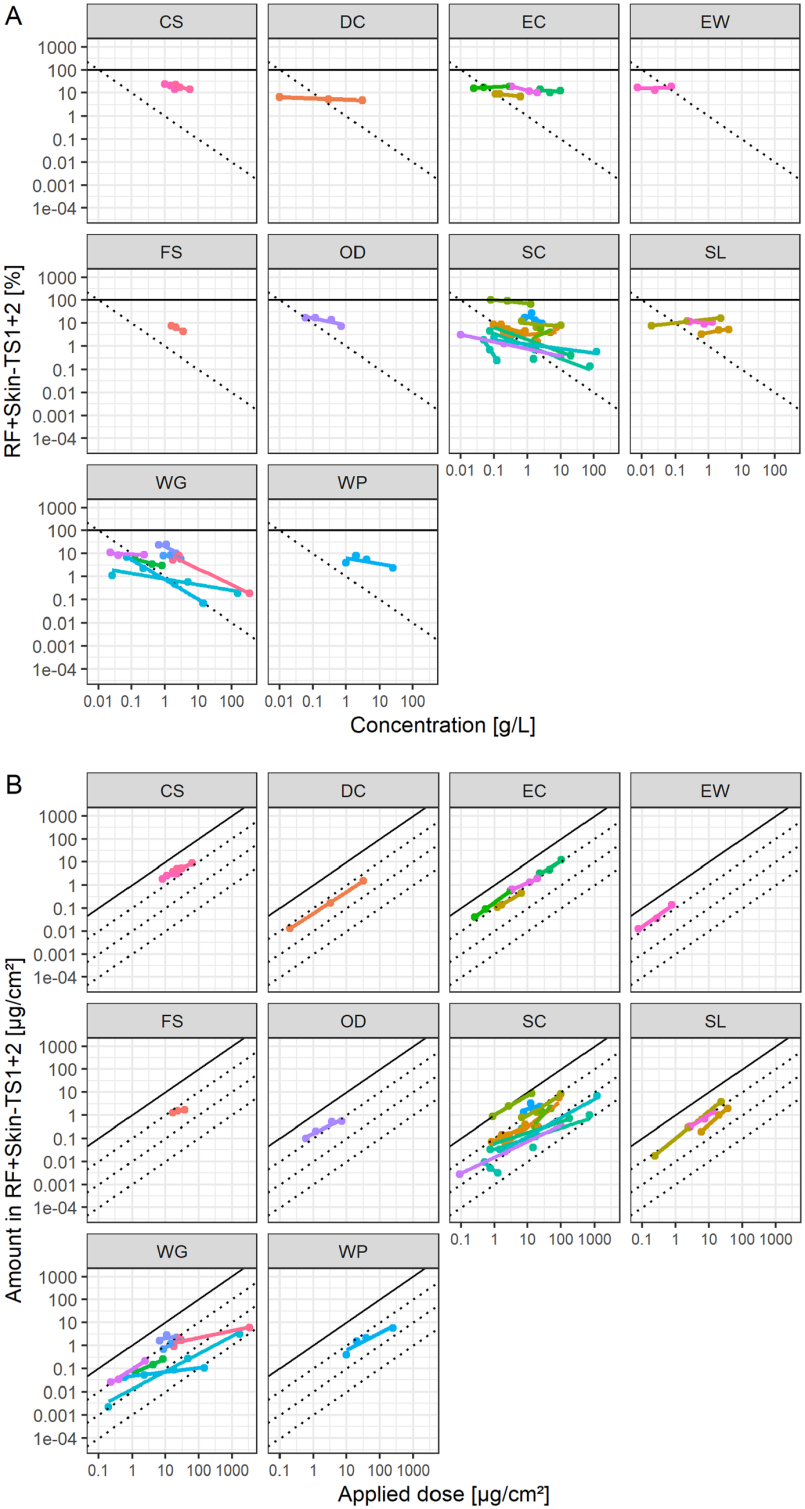
Potentially absorbed dose of CLE collected mean data of studies conducted with multiple concentrations of in-use dilutions, expressed as **A** relative absorption and concentration or **B** applied dose, latticed by formulation type. Linear models were fitted per study, colored by study

Hence, CLE collected dermal absorption in vitro data where at least 3 concentrations were tested for in-use dilutions, to explore the hypothesis of a direct relationship of applied dose and absorbed dose. Figure 4 shows the penetration based on receptor fluid values and Fig. 5 based on potentially absorbed dose, each line represents a linear model fit per tested product in-use dilution. Overall model fits for relative absorption and concentration have slopes of –0.32 and –0.25 and *R*^2^s of 0.10 and 0.13 for receptor fluid recovery and potentially absorbed dose, respectively. For the absolute amount applied and penetrated, the fits have slopes of 0.68 and 0.76 and R^2^s of 0.35 and 0.59 for receptor fluid recovery and potentially absorbed dose, respectively. Again, the absolute values seem to characterize the absorption characteristics better than relative absorption and concentration. The models can also be investigated by comparing AIC (Table 2), but there is no difference on an average model basis. However, the dataset allows a consideration of the individual products as a random factor in a mixed-model, since multiple doses were tested. Here, the models with absolute values have lower AIC values than those using relative absorption and concentration, however, the difference is small. Comparing mixed models with *R*^2^ is challenging but can be achieved by calculating both marginal and conditional *R*^2^s, considering only the fixed or fixed and random effects together, respectively (Barton 2022; Nakagawa et al. 2017). Here, the models using absolute values perform consistently and substantially better than those based on relative absorption and concentration (Table 2), with marginal *R*^2^s more than doubling.

No data were available where multiple doses of product concentrate concentrations were tested.

In total 33 studies (of 32 products, one of which used two active ingredients) were collected, covering 10 different formulation types, *i.e.*, CS, DC, EC, EW, FS, OD, SC, SL, WG, WP, using international formulation codes (CropLife International 2017; WHO 2016).

Most of the linear model slopes are compatible (Hothorn and Pirow 2020; Kluxen 2020) with 1 when using a 95% confidence interval, *i.e.*, 25 of 33 (76%) based on receptor fluid and 24 of 33 (73%) based on potentially absorbed dose. Hence the data supports the hypothesis that absolute absorption directly correlates with applied dose.

Eight slopes are not compatible to 1, i.e., 2 CS, 1 EC, 1 FS, 3 SC, 1 WG.

**Fig. 6:**
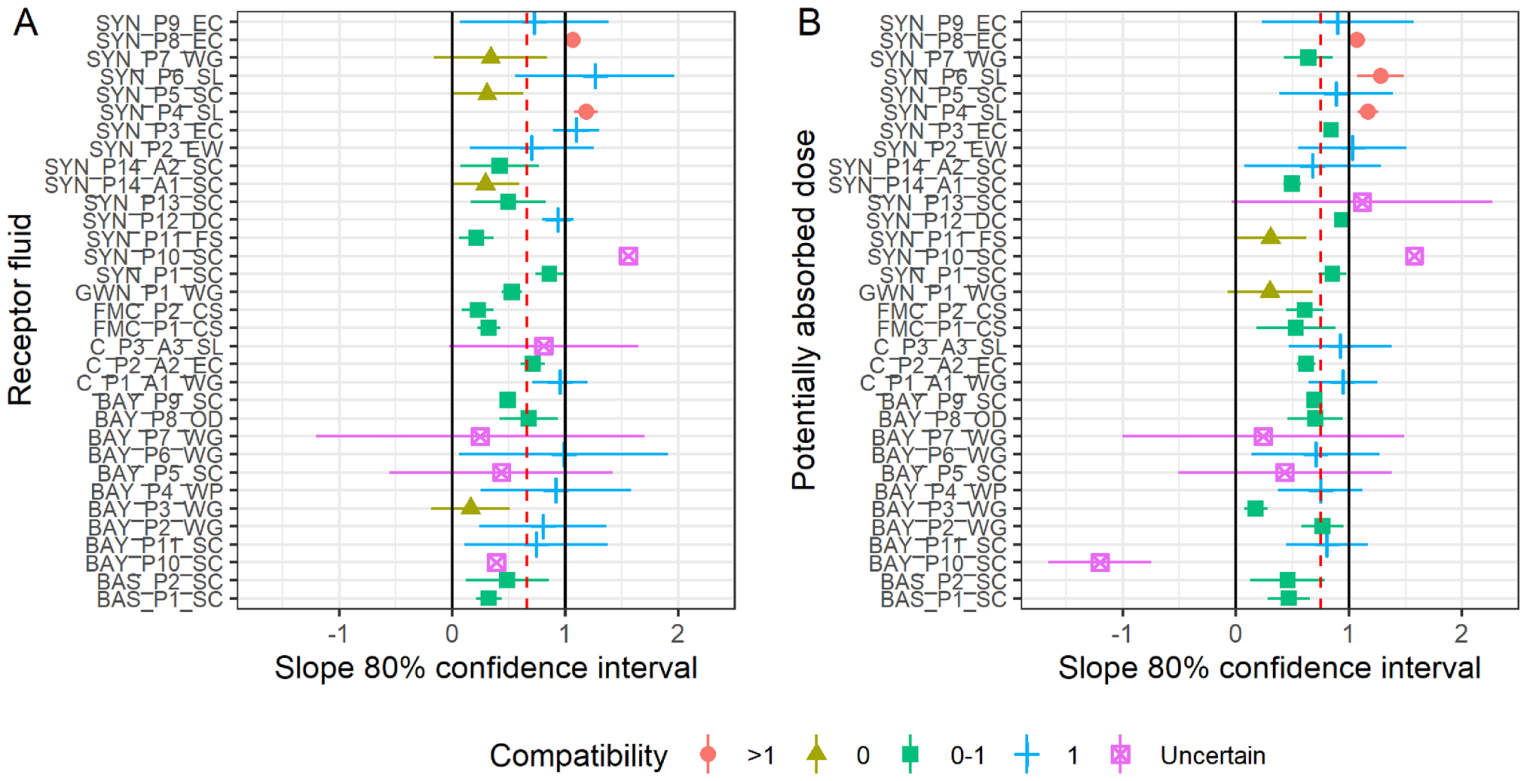
80% confidence intervals of the slopes of the fitted models to in use dilution data based on **A** receptor fluid recovery or **B** potentially absorbed dose. Color indicates compatibility with 4 hypotheses with 95% confidence in each. The negative slope of BAY_P10_SC, which is driven by *stratum corneum* residue, is classified as “uncertain”; the confidence intervals of SYN_P10_SC exceed the graph limits and are clipped (color figure online)

Many models that numerically deviate from a slope of 1 are only statistically compatible with 1 due to wide confidence intervals. This highlights a general issue associated with assessments that are only based on statistical assessments, compare Kluxen and Jensen (2021). Hence, rather than investigating compatibility with 1 and ignoring compatibility with 0, it is instructive to explore multiple hypotheses simultaneously, which can be also addressed with confidence intervals. Figure 6A shows 80% confidence intervals of the slopes of the fitted models based on receptor fluid recovery, here 4 hypotheses can be assessed with 95% confidence each (Table 3).

**Table 3:** Slope compatibility of 33 studies collected by CLE with multiple tested in-use dilution concentrations

Slope compatibility	Receptor fluid		Potentially absorbed	
	*n*	%^a^	*n*	%^a^
One hypothesis, 95% confidence intervals				
1	25	76	24	73
Multiple hypotheses, 80% confidence intervals				
0	4	12	2	6
0–1	12	36	14	42
1	10	30	9	27
>1	2	6	3	9
Uncertain	5	15	5	15

^a^Rounded

The confidence intervals of 5 studies are too large to make any conclusion. Two studies have slopes greater than but very close to 1, and 4 slopes are compatible with 0. 12 cases are compatible with a slope between 0 and 1 and also 10 are compatible with a slope of 1. Overall, the data are not in agreement with a slope of 0 hypothesis, which is the basis of the pro-rata approach used in European registration processes, when using in-use dilutions. A similar assessment can be made when the slopes of models considering the potentially absorbed dose is investigated (Fig. 6B).

It is probable that single products and actives deviate from a slope of 1 due to physical–chemical properties and associated interaction with the skin. For example, Fig. 4B shows the effect of including skin recovery in the dermal absorption estimation, and particularly how some responses are skewed, *i.e.*, by changing the slope to <0 for BAY_P10_SC. Here, lower doses are associated with a higher absolute amount absorbed, since *stratum corneum* residue was not removed by washing or due to a high skin association. It was previously argued that residue in the *stratum corneum* does not contribute to systemic dose (Aggarwal et al. 2014, 2015) and is not relevant for 24-h risk assessment (Kluxen et al. 2021). The log–log plot shows how the absorption relationship is skewed for some active ingredients when generically including *stratum corneum*. It also highlights that the resultant slope is biologically implausible, as reviewed in Table 1.

It needs to be highlighted that, while the statistical assessment shows that dose is a good descriptor of absolute absorption and that the pro-rata adjustment is not supported, about half (45%) of the slopes for the receptor fluid and almost a third (27%) for potentially absorbed dose are numerically below 0.5, *i.e.*, ignoring uncertainty in the slope estimate. Hence, there seems to be a substantial confounding effect by concentration, solubility, and co-formulants in this dataset, which could have been a priori expected based on the *status quo*. The corresponding products are both CS and the FS formulation, 9 SC and 3 WG for the receptor fluid and the FS, 5 SC and 3 WG for potentially absorbed dose. The formulation types may indicate solubility as the main driver of this effect.

However, a significant fraction is very close to a slope of 1, *i.e.*, about 20% of the slopes are within [0.9, 1.1] and 30% and 36% within [0.8, 1.2], for receptor fluid and potentially absorbed dose, respectively, and thus concentration, solubility, and co-formulants have only a negligible effect for this fraction of the dataset.

### Triple pack studies

**Fig. 7:**
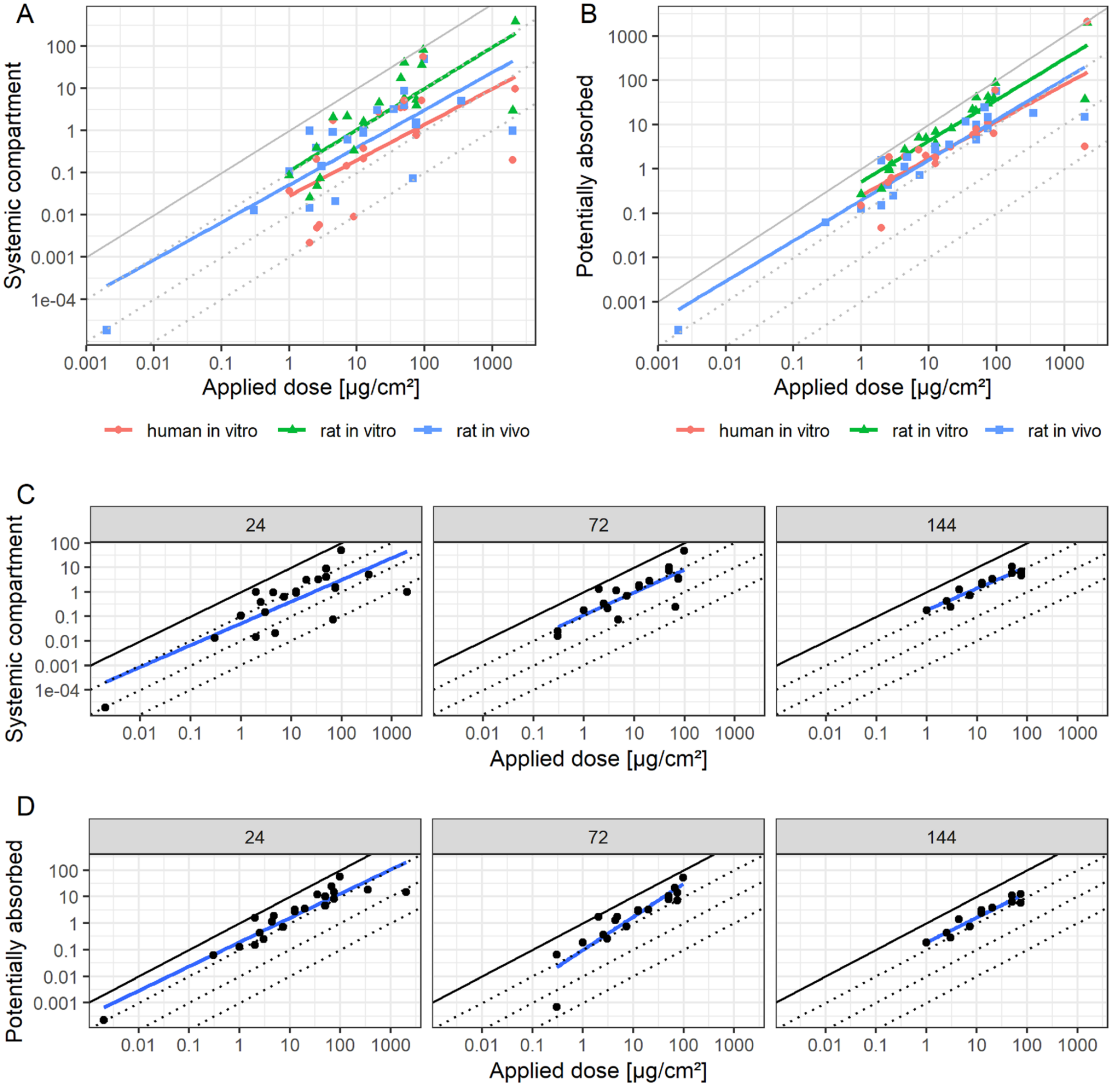
CLE collected mean in-use dilution data of triple pack studies, in vitro human (red)*, *in vitro rat (green) and in vivo rat (blue), expressed as applied dose for **A** the systemic compartment and **B** potentially absorbed dose. Linear models were fitted per study type. And, in vivo rat study mean data by most common observation time point, expressed as applied dose for **C** the systemic compartment and **D** the potentially absorbed dose. Linear models were fitted per observation time point (color figure online)

CLE further collected and evaluated 15 triple pack datasets. The in-use dilution data are shown in Fig. 7A, B, where each line represents an overall linear model fit per test type, *i.e.*, in vitro human (red), in vitro rat (green) and in vivo rat (blue). It shows that the linear regression models are very close to 1, most closely for the in vivo data. All models are compatible with a slope of 1 for systemic dose/receptor fluid and 2 of 3 models for potentially absorbed, using a 95% confidence interval. On average, the in vitro rat assay overestimates in vivo rat and results in higher absorption estimates than the in vitro assay using human skin. This relationship was also recently reported by Allen et al. (2021), which included almost all of the CLE data.

The in vivo data was also stratified by the most commonly assessed time points, *i.e.*, 24, 72 and 144 h (Fig. 7C, D). The plots show again a slope very close to 1 independent of the observation time point. Further, on average the systemic dose slightly increases over time (*n.b.*: the cumulative systemic dose), while the potentially absorbed dose remains constant. This seems to be driven by a flux from the *stratum corneum*, as linear models considering absolute absorbed doses and investigated time point show on average a negative slope (data not shown), which indicates that absorption is driven by a concentration gradient in the compartments.

Overall, also the in vivo/triple pack data supports a direct relationship of the applied dose.

### Calculating an average dermal absorption value

When the absolute dose is considered to be a good descriptor of absorption characteristics, an average dermal absorption value can be derived for an active ingredient in a formulated product, which is described in this section.

### Using a fitted linear model

Fitting a simple linear model on a log–log scale allows the derivation of an average dermal absorption estimate, which is characterized by the *y*-intercept.

The model is characterized by the following formula:

$${\text{mean}}\left( {\log 10\left( {\text{absorbed dose}} \right)} \right) = {\text{ slope}} \times {\text{mean}}\left( {\log 10\left( {\text{applied dose}} \right)} \right) + {\text{intercept}}$$

The relative dermal absorption value based on the model (DA_rel|model_) is:

$${\text{DA}}_{{\text{rel}}|{\text{model}}} \left[ \% \right] \, = { 1}0^{{\text{log1}}0({\text{intercept}})} \times {1}00.$$

For the EFSA DA DB data (Table 4), this means that on average receptor fluid recovery of in-use dilutions, independent of formulation type, corresponds to DA_rel|model_ = 2.71% (2.69% based on the rounded values in Table 4) of the applied dose based on the model derived parameters.

**Table 4:** Average dermal absorption calculation based on a fitted model mean to dermal absorption data of the EFSA DA DB

Endpoint	Test item	Fitted model intercept	DA_rel|model_	DA_ave_
Receptor fluid	Concentrate	−2.29	0.51	0.18
	Dilution	−1.57	2.71	2.25
Receptor fluid + Skin – TS1/2	Concentrate	−1.78	1.64	0.84
	Dilution	−0.99	10.26	7.72

DA_rel|model_ = relative dermal absorption based on fitted model slope

DA_ave_ = averaged relative dermal absorption value assuming a slope of 1

### Simplified calculation assuming a slope of 1

Assuming a direct relationship of applied dose and absorbed dose, *i.e.*, a slope of 1, simplifies the calculation of the corresponding y-intercept by making model fitting redundant.

Rearranging the formula, the intercept, and thus the basis for the DA_rel_ calculation, given the slope is 1, is the mean of the difference of the decadic logarithms of absorbed dose and applied dose.

$${\text{Intercept }} = {\text{ mean}}\left( {{\text{log1}}0\left( {\text{absorbed dose}} \right) \, - {\text{ log1}}0\left( {\text{applied dose}} \right)} \right)$$

The corresponding average dermal absorption value (DA_ave_) can be derived by (back-)transformation to percentage scale.

$${\text{DA}}_{{\text{rel}}|{\text{slope}} = {1}} = {\text{ DA}}_{{\text{ave}}} \left[ \% \right] \, = { 1}0^{{\text{intercept}}} \times {1}00$$

The average receptor fluid recovery of in-use dilutions, independent of formulation type, accordingly corresponds to 2.25%, which is very similar to the model-based value because the fitted model slope is 0.9.

Compare the numerical example calculations in Table 5 containing data from dermal absorption studies, with different slopes. It shows that the calculation can be easily performed with any available software or by hand. The table also shows that the estimated internal dose based on DA_ave_ is higher on average than the one based on relative DA values for slopes < 1. This aligns with the toxicological axiom of dose driving toxicity/risk through internal exposure.

**Table 5:** Example calculation of DA_ave_, assuming a slope of 1 and comparison of internal dose estimates based on DA_rel_ and DA_ave_, and based on the receptor fluid recovery

Product	Linear model slope	Study data means		Applied × DA% = absorbed dose	Log10		Difference (absorbed-applied)	Mean difference	DA_ave_ (%)^a^	Absorbed dose using DA_ave_
		Applied dose (µg/cm^2^)	DA (% RF)		Applied dose	Absorbed dose				
FMC_P2_CS	0.227	62.37	5.2	3.24	1.79	0.51	−1.28	−1.02	9.50	5.92
		31.43	8.93	2.81	1.50	0.45	−1.05			2.98
		22.7	12.54	2.85	1.36	0.45	−0.90			2.16
		16.46	13.96	2.30	1.22	0.36	−0.86			1.56
	Sum	132.96		11.19						12.62
BAY_P6_WG	0.987	29.7	1.32	0.39	1.47	−0.41	−1.88	−1.84	1.46	0.43
		14.8	1.8	0.27	1.17	−0.57	−1.74			0.22
		8.8	1.3	0.11	0.94	−0.94	−1.89			0.13
	Sum	53.3		0.77						0.78
SYN_P10_SC	1.56	49.8	2.54	1.27	1.70	0.10	−1.59	−1.67	2.14	1.07
		26.2	3.0	0.79	1.42	−0.10	−1.52			0.56
		16.5	1.29	0.21	1.22	−0.67	−1.89			0.35
	Sum	92.5		2.27						1.98

^a^DA_ave_ [%] = 10^mean difference^ × 100

## Discussion

According to the presented data and toxicological philosophy, the model of absolute absorption depending on applied dose seems to be a reasonable alternative or even superior model to relative absorption depending on applied concentration. Both models describe some aspects of a test item’s dermal absorption properties, and the models are obviously highly correlated. Still, the level of increased model performance with regard to correlation and explained variance is encouraging.

The proposed approach offers new research opportunities. It could be systematically investigated how concentration and dose interact, which could be used to substantiate a revision of regulatory assumptions such as the pro-rata approach in Europe or improve computational tools for dermal absorption prediction. The current data consist only of regulatory studies that do not allow to investigate such an interaction directly. With respect to the CLE data, one needs to consider, that dermal absorption studies are generally only conducted if there is a regulatory incentive, *i.e.*, to refine a risk assessment. Products with studies with multiple tested concentrations may have been especially challenging products for risk assessment, *e.g.*, due to complex interactions with solubility or low reference values, which may not be representative for most used agrochemical formulations.

Usually, dermal absorption is very generally characterized by Fick’s laws, *i.e.*, there is a flux over a gradient between different concentrations. These underlying physical–chemical processes in dermal absorption studies are usually ignored in the regulatory application of such studies, which use the final cumulative amount expressed as relative absorption. However, one can use the observed maximum flux in a study to abstractly characterize test item penetration behavior, which could also be used in risk assessment by translation into absorption over time. The flux approach ignores skin residues, which would overcome many regulatory issues for low-penetrating compounds, where the skin residue does not relevantly contribute to the surrogate systemic dose but needs to be accounted for in some regulatory areas. The flux approach is seldom accepted by European authorities, albeit promoted for years by risk assessors and recently considered by individual member state authorities (*personal communication*). Thus, the approach presented in this paper relates to the current regulatory de facto standard of using relative values.

Contrary to the relationship described by Fick’s laws, the actual penetration in the dermal absorption process is more complex (Dragićević and Maibach 2021). The skin consists of multiple heterogeneous layers and the labeled material is within a product and/or its spray dilution. Accordingly, there are many competing diffusion processes, *e.g.*, due to lipophilicity, occurring over the set of media or compartments in a dermal absorption cell. The complexity of this system may explain why there are test items where the slope of 1 assumption does not hold. The solid test items, WG (Water Dispersible Granules) and WP (Wettable Powder) are interesting. In Table 5, the WG example has an actual slope of 0.987; other WGs have slopes < 0.5 and the lowest is 0.16. For solid test items, the reasons may be associated with solubility and drying processes, because the test items are applied as slurries for the higher concentrations, *i.e.*, concentrated formulations. Those processes most likely interact.

Regulatory dermal absorption studies use fixed applied volumes, *e.g.*, 10 µL/cm^2^, which was considered to represent typical exposure scenarios against droplets, in the development of OECD TG 428. Hence, there is a relationship of applied dose and concentration; in the CLE dataset the Pearson correlation coefficient is indeed 0.9988. However, it is surprising that the very sparse model of describing absorbed dose only by applied dose covers much of the observed variation and more than concentration. A large fraction of products, for which a model slope is compatible with 1, absorption is neither critically dependent on the applied concentration nor on other factors. Hence, the data are also in stark conflict with the EFSA pro-rata assumption, where a relative absorption value is derived by pro-rata extrapolation for a more diluted concentration than those tested. Thus, we believe that the dose as the driver of absolute absorption is a reasonable model, which is supported by the data.

The key issue of using dermal absorption factors in non-dietary risk assessment in Europe is that the factor is considered based on tested concentrations and not on applied doses on the skin. This results in different dermal absorption estimates between Europe and other regulatory areas, and disconnects exposure dose from dermal absorption, with associated challenges for risk assessment. Another developing field on quantitative risk assessment, *i.e.*, contact to skin sensitizers contained in pesticidal products (Sanvido et al. 2018), is also struggling with the disconnect of dermal absorption information and the actual quantification of exposure at the site of contact relevant to elicit the assumed hazard. The relevant dose-metric applied in quantitative risk assessment of skin sensitizers is the dose per unit area of skin (µg/cm^2^) forming the basis in routine skin sensitization risk assessment for fragrances (Api et al. 2008, 2020; Kimber et al. 2008). Further, one may argue that the current risk assessment approaches for pesticides in Europe and other regulatory regions incrementally but fundamentally impact the understanding of Fick’s laws in the regulatory community, as the approaches consider the dermal absorption as a relative factor in terms “percentage of applied dose”. The thereby noticed so-called inverse concentration or dose-relationship may misleadingly suggest that Fick’s laws are incorrect and diffusion is not directly proportional to the concentration gradient or applied dose but is instead inversely proportional, which is just an artifact from using a relative factor.

The current approach for non-dietary risk assessment binds resources because the assessment is complicated and time consuming. It also results in very conservative absorption estimates, as it compounds conservatism already built into the assay design (Kluxen et al. 2021), which then often triggers further studies that also need to be incorporated in exposure assessments and risk evaluations.

It is thus proposed to utilize dermal absorption data differently by deriving an average dermal absorption estimate that can be used independent of dose or concentration instead of considering dermal absorption in relation to applied concentration. An alternative view could be that the method allows deriving a single product-specific default dermal absorption value. This may also allow for a more balanced comparison of products considering relative dermal absorption as an inherent product property, which is not biased by a specific tested concentration but averaged over a concentration range.

A relationship of applied dose and absolute amount absorbed has been also recently and independently described by a project of *Cosmetics Europe* (Hewitt et al. 2020), expressed as receptor fluid and skin excluding *stratum corneum* recovery. Here, 56 cosmetic-relevant chemicals have been tested according to OECD TG 428, a linear model fitted to the log-transformed data resulted in an *R*^2^ of 0.47. We see a similar overall relationship in both the EFSA DA DB and the CLE data with multiple tested concentrations for agrochemical formulations that also contain co-formulants and are thus mixtures, as compared to the results from Hewitt et al. (2020) that tested individual chemicals.

If a slope is 1 in the proposed model, dermal absorption can be assumed to be constant for a product and independent of the applied dose (Table 1). This results in the practical implication that a single dermal absorption value is appropriate independent of exposure dose. While no product concentrate data was available with multiple doses tested, one could expect that concentrates would behave similarly, based on the EFSA DA DB concentrate data.

In addition to the solid test items, some other slopes in the in vitro CLE dataset are noticeable below 1, which was also supported by the refined analysis using 80% confidence intervals. Hence, there are products where absorption changes disproportionally with applied dose; the reasons for this are unclear for the in vitro assay but may be also be associated with physical–chemical processes not specifically investigated in regulatory studies. Hypothetically, since agrochemical products are intended for application in an in-use dilution, the co-formulants may incidentally affect the optimal environment for penetration, either by decreasing penetration at higher concentrations or vice versa. Only some agrochemical co-formulants are intended to increase the bioavailability to plants and often with plant-specific targets, for example, the waxy cuticle, hence, there does not necessarily have to be a relationship of co-formulants and dermal penetration properties. For some chemicals, the effect may be related to a chemical’s properties or assay design and not necessarily with mixture partners. It may for example also be associated with insufficient washing, as the concentration gradient between wash solution and skin residue decreases with a reduced applied dose, which may affect standard washing efficiency. Conversely, lower doses may generally also be more susceptible to a wash-in effect (Moody and Maibach 2006) or products could generally differ in their susceptibility to the wash-in effect, especially if studies are conducted in different laboratories with different standard approaches. This may also interact with exposure duration, which may vary according to the OECD test guideline and should only be representative for the risk assessment, e.g., cover a typical workday exposure scenario of 6–10 hours. It was recently shown that the harmonization of study parameters leads to very compatible in vitro dermal absorption study outcomes independent of the performing laboratory (Kluxen et al. 2022). Further, evaporation or drying of the test item can affect dermal absorption (Hewitt et al. 2020). Finally, also concentration can be expected to affect in vitro kinetics to some extent and thus the cumulative amount absorbed after 24 h, however, the impact seems to vary by product tested, for products with a resulting fitted slope close to 1, other factors than dose seem to be of negligible impact. Some test items are only suspended in the in-use dilution and may thus have similar properties as solid test items—hence, the amount of solubilized test item may change by concentration. However, such processes need to be evaluated within the exposure scenario that the studies are considered to model, see below.

It is per se plausible that systemic burden does not directly correlate with external dose due to ADME effects; in practice, the material penetrating into the systemic compartment is not equal to the applied amount, *e.g.*, due to metabolism. Further, the in vitro assay and cumulative assessment does not consider excretion, which is usually demonstrated in regulatory in vivo studies for the registered active ingredient. However, the question is how to leverage such information for risk assessment purposes. It might make sense to balance cumulative in vitro dermal absorption assays with excretion assays to better estimate systemic burden.

When a slope is below 1, assuming direct absorption proportionality to applied dose, *i.e.*, a slope of 1, underpredicts the dermal penetration estimate for lower doses, but overpredicts the dermal absorption estimate for higher doses. From an exposure point of view this leads to a more conservative assessment since the exposure dose drives risk and not dermal absorption.

For many scenarios, the exposure doses are nominally low, *e.g.*, for re-entry activities. Thus, the proposed method may raise concerns from risk assessors as being insufficiently protective. However, as described above, in a functional biological system, the systemic concentration of a test item is dynamic as a function of at least penetration and excretion. Hence, the effect on small doses should be covered by the conservative nature of the assay system (Kluxen et al. 2021).

Further, exposure doses in re-entry activities such as harvesting or when handling equipment usually cumulate over the work day on relatively defined skin areas, *e.g.*, hands (Ross et al. 2000), due to contact with dried residues or due to droplets with peak and spot exposures. Hence, doses are not uniformly distributed as a low dose over the total body surface. This is also considered in exposure models, *e.g.*, the European worker re-entry risk assessment and could be used to determine relevant doses for dermal absorption testing (Morgan et al. 2020). Accordingly, assuming dermal absorption estimates for low doses and thus extrapolating the dermal absorption assay skin dose to an exposure scenario with higher locally defined doses will rarely result in realistic risk estimates, but conversely likely overpredict risk. However, even if such evenly distributed low skin doses would occur in an exposure scenario, very small doses are more likely to be remaining in and being desquamated via the *stratum corneum,* do not penetrate simultaneously from the different exposure areas, due to barrier and thus kinetic differences between skin areas (Bormann and Maibach 2020; Dragićević and Maibach 2021), and are less likely to exceed ADME detoxification capacity than higher doses*.* Hence, the assay with the inbuilt conservatism becomes increasingly unrealistic for risk assessment of very small exposure doses. Thus, again an overprediction of the dermal penetration estimate for the higher applied doses appears to be protective.

When a slope is exceeding 1, assuming absorption proportionality to applied dose, *i.e.*, a slope of 1, overpredicts the dermal absorption estimates for lower doses but underpredicts the dermal penetration estimate for higher doses. The available data do not indicate that such a case is likely or common. The observed cases in the CLE dataset were very close to 1, and could be likely driven by experimental variation, with only one exception. However, assuming a slope of 1 in such cases may not be considered sufficiently conservative for risk assessment purposes, as risk may be underpredicted by a single dermal absorption value. Hence, it is suggested to not generically assume a slope of 1 in such cases. Conversely, it is toxicologically plausible that a slope > 1 exists for systemic exposure against the active ingredient, compared to Table 1. It implies that ADME processes reduce systemic exposure and that it limits to 0% absorption for very small doses. Hence, a DA_ave_ should be also protective when the slope is > 1.

Based on the presented data, dermal absorption can be estimated over the full range of possible exposure doses by assuming a slope of 1 in a log–log regression model. This approach has several benefits.

- It directly connects the doses tested in dermal absorption studies with estimated doses in exposure models or measured values from exposure studies.
- It simplifies exposure estimation as the in-use dilution sprayed in a scenario can be disregarded—the in-use dilution concentration is often not relevant for risk assessment, *e.g.*, for re-entry scenarios and designing exposure-driven dermal absorption studies might be challenging (Morgan et al. 2020).
- Uses with dilutions outside of the tested range can be assessed without generating new dermal absorption data.
- The approach is conservative because dermal absorption estimates for higher doses are increased when the fitted slope is < 1.

A typical dermal absorption study set up could thus comprise testing at least 3 doses and a linear model fit on log–log scale. If the model’s slope is ≤ 1, *e.g.*, based on a visual assessment or the slope’s confidence interval, dermal absorption can be considered to be constant for a product and not depending on the applied dose. Hence, a single dermal absorption value can be used for all exposure scenarios. If the model slope exceeds 1, this should be dealt with case-by-case, maybe utilizing kinetic data from ADME studies available on the active substance.

Concentrates and in-use dilutions have vastly different physical–chemical properties, which results in overall different dermal absorption estimates (Aggarwal et al. 2014, 2019). Hence those should be tested separately and not incorporated in the log–log dermal absorption model. However, the in-used dilution value should be suitable for read-across to its respective concentrated product.

Overall, the presented approach is simple and can be readily implemented in existing or novel risk assessment practice.

## Conclusion

In vitro and in vivo dermal absorption data indicate that absolute absorption is directly dependent on applied dose, which may be a suitable alternative or even superior model to considering that relative absorption is only dependent on concentration. This allows a derivation of a single dermal absorption estimate, which greatly simplifies the use of dermal absorption data in risk assessment when data on multiple concentrations are available.

The relationship between concentration and applied dose in dermal absorption assays conducted with agrochemical formulations warrants further investigation, however, the current manuscript supports a new model for interpretation and use of dermal absorption data.
